# Tracking the Re-organization of Motor Functions After Disconnective Surgery: A Longitudinal fMRI and DTI Study

**DOI:** 10.3389/fneur.2018.00400

**Published:** 2018-06-05

**Authors:** Cristina Rosazza, Francesco Deleo, Ludovico D'Incerti, Luigi Antelmi, Giovanni Tringali, Giuseppe Didato, Maria G. Bruzzone, Flavio Villani, Francesco Ghielmetti

**Affiliations:** ^1^Neuroradiology Unit, Fondazione IRCCS Istituto Neurologico “Carlo Besta”, Milan, Italy; ^2^Scientific Department, Fondazione IRCCS Istituto Neurologico “Carlo Besta”, Milan, Italy; ^3^Clinical Epileptology and Experimental Neurophysiology Unit, Fondazione IRCCS Istituto Neurologico “Carlo Besta”, Milan, Italy; ^4^Health Department, Fondazione IRCCS Istituto Neurologico “Carlo Besta”, Milan, Italy; ^5^Neurosurgery Unit, Fondazione IRCCS Istituto Neurologico “Carlo Besta”, Milan, Italy

**Keywords:** fMRI, DTI, motor functions, epilepsy, disconnective surgery, cortico-spinal tract

## Abstract

**Objective:** Mechanisms of motor plasticity are critical to maintain motor functions after cerebral damage. This study explores the mechanisms of motor reorganization occurring before and after surgery in four patients with drug-refractory epilepsy candidate to disconnective surgery.

**Methods:** We studied four patients with early damage, who underwent tailored hemispheric surgery in adulthood, removing the cortical motor areas and disconnecting the corticospinal tract (CST) from the affected hemisphere. Motor functions were assessed clinically, with functional MRI (fMRI) tasks of arm and leg movement and Diffusion Tensor Imaging (DTI) before and after surgery with assessments of up to 3 years. Quantifications of fMRI motor activations and DTI fractional anisotropy (FA) color maps were performed to assess the lateralization of motor network. We hypothesized that lateralization of motor circuits assessed preoperatively with fMRI and DTI was useful to evaluate the motor outcome in these patients.

**Results:** In two cases preoperative DTI-tractography did not reconstruct the CST, and FA-maps were strongly asymmetric. In the other two cases, the affected CST appeared reduced compared to the contralateral one, with modest asymmetry in the FA-maps. fMRI showed different degrees of lateralization of the motor network and the SMA of the intact hemisphere was mostly engaged in all cases. After surgery, patients with a strongly lateralized motor network showed a stable performance. By contrast, a patient with a more bilateral pattern showed worsening of the upper limb function. For all cases, fMRI activations shifted to the intact hemisphere. Structural alterations of motor circuits, observed with FA values, continued beyond 1 year after surgery.

**Conclusion:** In our case series fMRI and DTI could track the longitudinal reorganization of motor functions. In these four patients the more the paretic limbs recruited the intact hemisphere in primary motor and associative areas, the greater the chances were of maintaining elementary motor functions after adult surgery. In particular, DTI-tractography and quantification of FA-maps were useful to assess the lateralization of motor network. In these cases reorganization of motor connectivity continued for long time periods after surgery.

## Introduction

Functional imaging techniques such as functional MRI (fMRI) and Diffusion Tensor Imaging (DTI) can track the structural and functional changes occurring during recovery after brain damage or surgery. In patients candidate to disconnective surgery, functional imaging can be used not only to localize motor activity, but also to investigate the mechanisms of motor plasticity observed before and after surgery. Disconnective surgery is used to treat drug-refractory hemispheric epilepsy caused by extensive congenital or acquired lesions, and provides seizure control in 60–90% of cases (1–5). Before surgery, patients often show residual function of their paretic limbs (6–8). After surgery, motor functions remain unchanged in most cases (50–60%), while in a lower percentage of patients it may deteriorate or even improve (9–11). Motor deterioration typically affects the upper limbs more than the lower limbs, while improvement can be more evident in children than in adults (12).

In patients with hemispheric or multilobar brain lesions it is not always clear from conventional MRI which hemisphere is associated with the residual motor ability of the paretic limb, and imaging techniques such as fMRI, DTI and Transcranial Magnetic Stimulation (TMS) can be useful to assess the integrity of sensori-motor network and cortico-spinal tract (CST).

The movement of the paretic limb can be mediated by the contralateral affected hemisphere, or the ipsilateral unaffected hemisphere or bilaterally. In a TMS and fMRI study of seven patients with hemiplegic cerebral palsy, the paretic hand was controlled by the unaffected hemisphere in four cases and bilaterally in three cases (13). In a TMS study of twelve patients with congenital hemiparesis for lesions in the periventricular white matter, Staudt et al. showed that large lesions and severe hand motor impairment were associated to ipsilateral projections originating from the unaffected hemisphere, while small lesions and mild hand motor impairment were associated to intact contralateral projections (6). However, the pattern of fMRI activations during paretic hand movement was similar for all patients, involving more the affected than the intact sensorimotor areas.

A number of imaging studies have investigated patients after hemispherectomy. fMRI showed that movement of the paretic limb was associated with controlesional activations of non-primary motor (SMA and premotor cortex) and sensory areas. This finding supports the hypothesis that after surgery the associative regions of the intact hemisphere are responsible for the execution of paretic limb movement (14–16). Postsurgical DTI revealed no evidence of reinforcement of the unaffected CST after hemispherectomy (17, 18).

Imaging techniques have been applied also in the preoperative state. DTI measures such as fractional anisotropy (FA) appear useful to assess residual integrity of CST before hemispherectomy and predict motor outcome after surgery (19). In particular, a DTI study with 102 patients showed that the CST asymmetry observed with a visual analysis of directionally-encoded color FA maps was useful to predict hand function (i.e., grasping ability) after hemidisconnection (19). A recent study employing fMRI and DTI showed that sensorimotor fMRI and colored FA-DTI maps could predict severe motor decline in a series of 25 patients (20). Only a few studies have investigated patients before and after hemispherectomy. fMRI and DTI performed in small groups of cases, undergoing different types of surgery, showed that the degree of re-organization of motor functions could vary according to the patient's pathology and surgery performed (21, 22). This is not unexpected, especially if surgery did not remove the motor cortex, and motor function could maintain part of its organization (22).

Evidence linking motor function to pre- and postoperative assessment is therefore rather limited. In this study we describe the residual motor functions of four adult patients candidate to a disconnective surgery of the fronto-central region, assessed clinically, with fMRI and DTI, and studied also after surgery. Patients were homogeneous for timing of lesions (before 2 years), timing of surgery (~30 years) and type of surgery which removed motor areas and disconnected the CST from the affected hemisphere. This allowed us to explore the mechanisms of motor reorganization occurring after the first remote insult, and how these re-adapted after adult surgery. A systematic analysis of arm and leg fMRI activations and DTI-derived FA-maps was performed before and after surgery with follow-ups of up to 3 years, in association with a semi-quantitative clinical scale. We hypothesized that preoperative lateralization of motor circuits assessed with fMRI and DTI in these four patients was useful to understand their motor outcome.

## Methods

### Subjects

Four patients (age range 28–39) with early brain lesions were studied between 2012 and 2016 at the Fondazione IRCSS Istituto Neurologico Carlo Besta, Milano. Patients underwent a tailored disconnective surgical procedure with partial resection of fronto-central areas involved in the origin and propagation of seizures (see Table 1 for characteristics). Muscle strength of the affected upper and lower extremities was assessed with the Medical Research Council Scale [MRC, (23)], before surgery and at 6–36 months post-surgery (Table 2). In this six-point scale, Grade 5 indicates normal muscle strength, grade 4 reduced power, grade 3 active movement against gravity, grade 2 active movement when gravity is eliminated, grade 1 trace of contraction, and grade 0 no detectable contraction.

**Table 1:** Clinical information of the four patients.

**Patient**	**Age range at surgery**	**Etiology**	**Onset of disease**	**Onset of epilepsy**	**Type of surgery**	**Seizure outcome Engel (follow-up)**	**Affected side**
Case 1	25–30	Measles encephalitis	1 year	5 years	Right frontal lobectomy and disconnection of the anterior two thirds of the corpus callosum	Ia (12 months)	L
Case 2	30–35	Severe head injury	6 months	9 months	Right fronto-central cortectomy and disconnection of fronto-central areas	Ia (34 months)	L
Case 3	35–40	Perinatal anoxia	0	6 years	Left frontal lobectomy and disconnection of about half of the corpus callosum	Ia (49 months)	R
Case 4	25–30	Ischaemic stroke	2 years	12 years	Left fronto-parietal cortectomy and fronto-parietal disconnection	IV (33 months)	R

**Table 2:** Motor performance at MRC scale before and after surgery.

	**Case 1**		**Case 2**		**Case 3**		**Case 4**	
	**PRE**	**POST (12 m)**	**PRE**	**POST (18 and 36 m)**	**PRE**	**POST (36 m)**	**PRE**	**POST (6 and 12 m)**
**UPPER LIMB**								
Shoulder abduction	4–	2	4–	*u*.	4	*u*.	4	*u*.
Shoulder adduction	4	3	4	*u*.	4+	*u*.	4	*u*.
Biceps flexion	4–	2	4–	*u*.	4+	*u*.	3	*u*.
Triceps extension	4–	1	3	*u*.	4	*u*.	3	*u*.
Wrist flexion	3	2	2	*u*.	3	*u*.	0	*u*.
Wrist extension	3	2	2	*u*.	3	*u*.	0	*u*.
Finger movements	0	0	0	*u*.	0	*u*.	0	*u*.
**LOWER LIMB**								
Thigh abduction and adduction	4+	4	4–	*u*.	5	*u*.	4	*u*.
Leg extension	4–	4–	4–	*u*.	4	*u*.	5	*u*.
Leg flexion	4–	4–	4–	*u*.	4–	*u*.	5	*u*.
Ankle flexion	4–	3	4–	*u*.	4–	*u*.	1	*u*.
Ankle extension	3	2	4–	*u*.	3	*u*.	1	*u*.

“*u.” refers to unchanged performance with respect to the preoperative assessment*.

Different groups of healthy subjects with no history of neurological or psychiatric disease participated in the study. For the fMRI hand motor task, 12 healthy participants were recruited (median age 37 years, range 25–54, 6 females, all right handed), while for DTI 15 participants (median age 34 years, range 29–60, 7 females, all right handed) were included. Imaging and clinical data were acquired and managed according to standard clinical procedures approved by the Research Ethic Board of the Institute (25/9/08, report N 17/ excerpt 26), and informed written consent was obtained from all participants.

### MRI data acquisition

Patients were imaged on a 3.0 T scanner (Achieva TX, Philips BV, Best, NL) using a 32-channel head coil, except for Case 2 who was imaged on a 1.5 T scanner (Magnetom Avanto, Siemens AG, Erlangen, DE) only for the preoperative session, using an 8-channel head coil. Head movement was minimized with decompression cushions. At 3.0 T, 140 volumes were acquired through an axial gradient-echo echo-planar imaging (EPI) sequence having TR= 3,000 ms, TE = 35 ms, 40 slices and voxel size 1.88 × 1.88 × 2.5. At 1.5 T, 200 volumes were acquired through an axial gradient-echo EPI sequence having TR= 2,000 ms, TE =45 ms, 26 slices with 3.5 mm isotropic voxel size.

The fMRI tasks were presented visually in a blocked-design with 12 active blocks (6 for each side) and 6 rest blocks. For the hand/arm motor task, patients repetitively raised their arm toward the shoulder, opening and closing their hand if possible, with a frequency of ~1 Hz. For the foot/leg motor task, patients raised the leg, alternating foot flexion and extension if possible. All patients practiced the motor tasks before scanning and performance was monitored visually during data acquisition. fMRI data analysis was performed with Brain Voyager (Brain Innovation, Maastricht, The Netherlands). After movement and slice timing correction, images were co-registered with the corresponding anatomical scans and smoothed using an isotropic Gaussian kernel (FWHM 4 mm). A single subject analysis was performed, based on the convolution of task boxcars with the canonical hemodynamic response function, with removal of movement-related nuisance variance. The resulting maps were thresholded with *p* < 0.05 false-discovery rate (FDR) or more, to get an adequate compromise between activation extent and presence of spurious clusters outside the motor cortex, a step reflecting common practice in presurgical settings (24, 25). Maps were then used in the preoperative assessment.

A Region-of-interest (ROI) analysis was performed for each patient on both healthy and affected hemispheres identifying (when possible): (i) the knob-like structure for the hand/arm motor area, (ii) the paracentral lobule for the foot/leg motor area, (iii) and for the Supplementary Motor Area (SMA) the paramedian area superior to the cingulate sulcus, anterior to the central sulcus, whose anterior boundary was defined by a line passing perpendicularly through the rostrum of the corpus callosum (26). ROIs were manually drawn with Freeview, part of the Freesurfer suite (27) on the basis of patient's anatomy and fMRI results, taking into account the high grade of anatomical distortions before and after surgery; in particular, an enlarged SMA was included. For each ROI, the number of voxels above threshold was computed. Symmetry of fMRI activations in the primary motor areas and SMA was determined by calculating the Laterality Index (LI) = (affected-unaffected)/(affected+unaffected).

DTI data were acquired with slightly different diffusion-weighted imaging sequences, as the study went on for several years. Common parameters are: TR/TE = 8,100/75, *b* = 1,000; 64 directions, voxel size = 2 × 2 × 2 (see Supplementary Table 1). Images were pre-processed for motion and distortion correction and whole brain deterministic fiber tractography was performed with ExploreDTI (28) running on Matlab 12 (The Mathworks, Natick MA, USA), with an angular threshold of 30° and a FA threshold of 0.2. Measures of FA, direction encoded color (DEC) map and tracts were computed and imported in TrackVis (www.trackvis.org). CST was traced on DEC map registered on the T1-weigthed image using as ROIs the anterior pons, the precentral gyrus, the post-central gyrus and the SMA (22, 29, 30). Placing an ROI in the anterior pons, which typically does not present DWI signal artifacts, FA values were also calculated in the z direction for a better assessment of fiber integrity along the direction of the tract. Symmetry in FA between ipsi- and contra-lesion CST was determined calculating the LI with the same formula used for fMRI.

## Case reports

### Case 1

**Figure 1:**
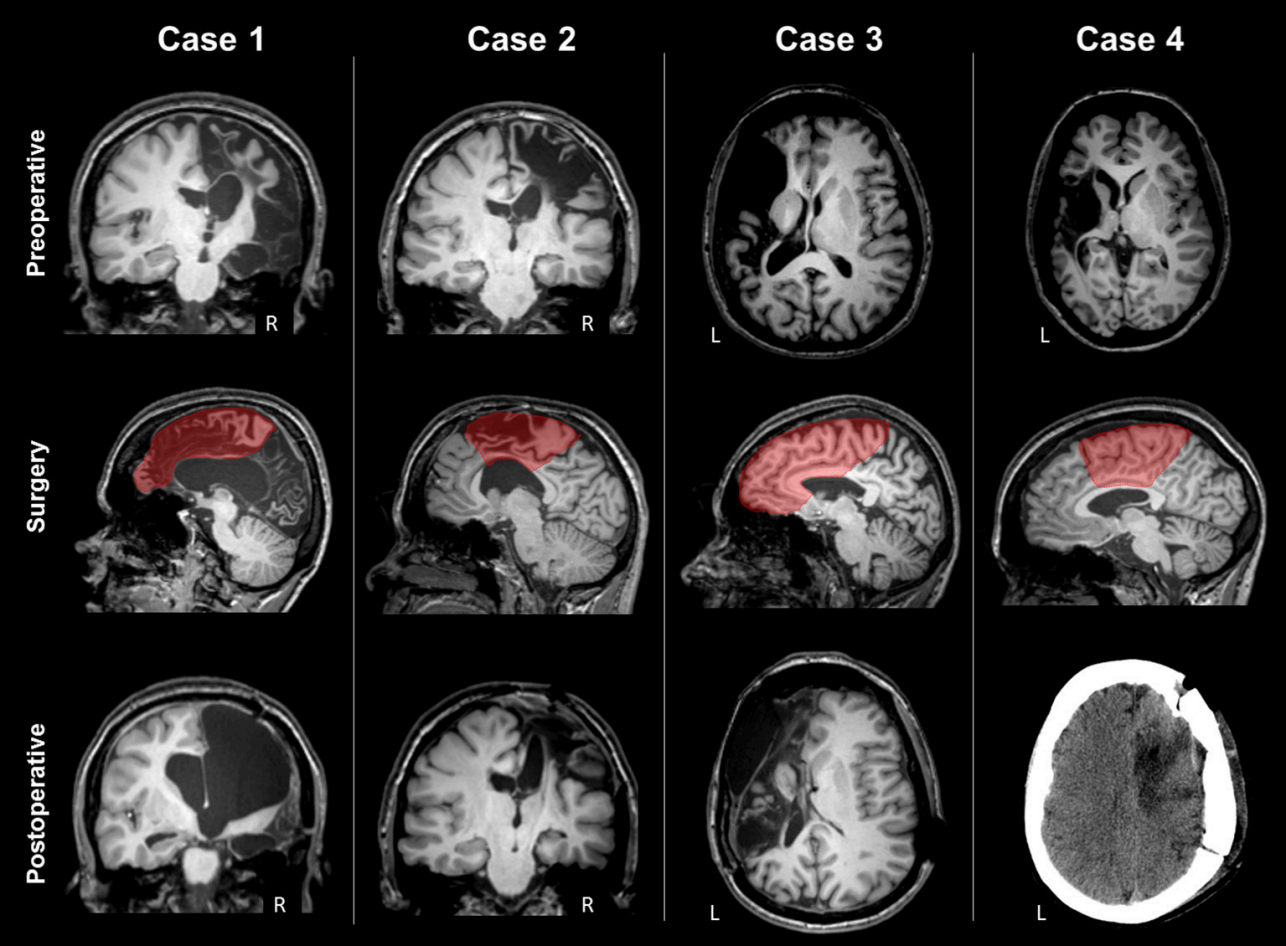
Preoperative MRI, type of surgery and postoperative scan of the four patients. Case 1 presented severe atrophy of the right hemisphere; rolandic convolutions were partially spared, and CST partially recognizable. The type of surgery was right frontal lobectomy. Postoperative MRI showed that the right CST was not recognizable anymore. Case 2 presented a right frontal malacic area partially sparing the motor cortex and the right CST. The type of surgery was a right fronto-central cortectomy with disconnection. Postoperative MRI showed removal of the upper part of motor cortex and degeneration of the right CST. Case 3 presented a large left frontal malacic area, sparing the upper part of the motor cortex, but involving the posterior limb of the internal capsule, and the CST. The type of surgery was right frontal lobectomy. After surgery, frontal convolutions were not recognized anymore. Case 4 presented a large left malacic area sparing the upper part of the motor cortex, but involving the internal capsule and the CST. The type of surgery was a right fronto-central cortectomy with partial disconnection, as observed in the postoperative CT scan.

The patient (age range: 25–30 years) suffered from measles encephalitis at the age of 1 year and developed drug-refractory epilepsy with left side tonic and clonic seizures at 6 years of age. She had left hemiparesis with limited hand grasping function and no fine finger movement (Table 2). MRI showed severe atrophy of the right hemisphere (Figure 1). The corpus callosum was severely atrophic, affected by the right hemispheric lesion and subsequent degenerative changes. Right frontal and rolandic convolutions were partially spared; CST and the posterior limb of the internal capsule were partially recognizable. The right cerebral peduncle was clearly atrophic. She underwent right frontal lobectomy and disconnection of the anterior two-third of the corpus callosum and of CST. After surgery the MRI showed removal of the residual frontal convolutions, including the motor cortex. The right CST and the internal capsule were not recognizable anymore. One year after surgery, the patient was seizure free (Engel class Ia). Motor function, assessed with MRC, worsened slightly for the lower limb, and more significantly for the upper limb (4 → 2 MRC scale).

#### fMRI

**Figure 2:**
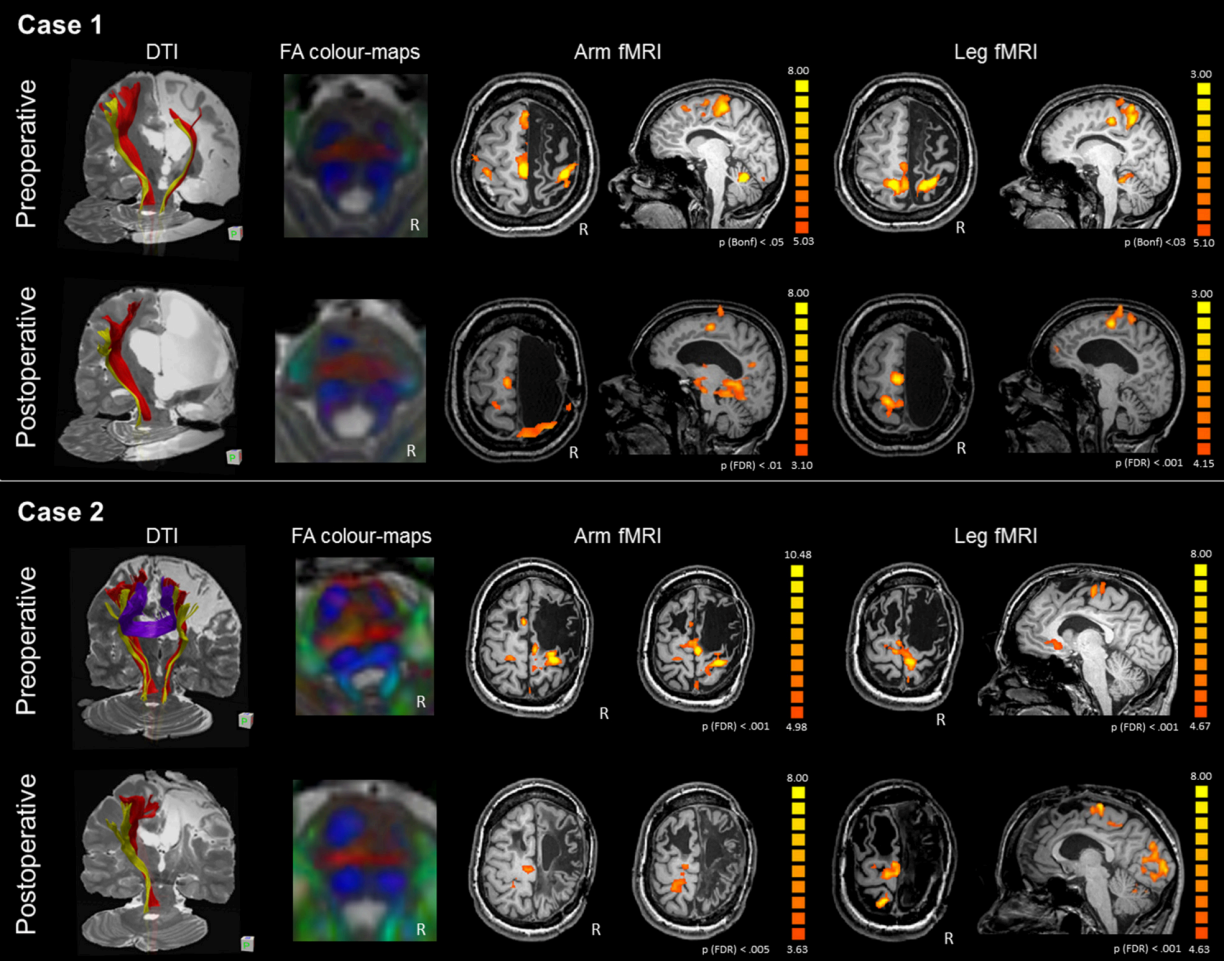
DTI-tractography and fMRI tasks of arm and leg movement of the preoperative and postoperative assessment of Case 1 and Case 2. Case 1 presented with severe atrophy of the right hemisphere and corpus callosum. DTI-tractography revealed the CST (red) and somatosensory tract (yellow) also in the affected hemisphere, while inter-hemispheric fibers were not reconstructed. After surgery the CST was no longer visible and the right somatosensory tract was recognized only between the brainstem and the thalamus. FA color-maps at the pons showed some asymmetry in the CST (anterior pair of blue) preoperatively, and a completely lateralized pattern postoperatively. Before surgery fMRI activations of the paretic arm and leg showed involvement of the right and left primary motor areas, with strong engagement of the intact SMA. After surgery, activations shifted to the intact hemisphere. Case 2 presented with a large frontal malacic area in the right hemisphere and a smaller one in the left hemisphere. DTI-tractography revealed the CST (red) and the somatosensory tract (yellow) also in the affected hemisphere, and inter-hemispheric fibers connecting homologous motor regions. After surgery, the right CST was no longer visible and the right somatosensory tract is recognized only between the brainstem and the thalamus. FA color-maps at the pons showed some asymmetry in the CST (anterior pair of blue) preoperatively, and a completely lateralized pattern postoperatively. Before surgery fMRI activations of the paretic arm and leg showed stronger involvement of the right than left primary motor areas, with engagement of the intact SMA. After surgery, activations shifted to the intact hemisphere, involving peri-rolandic areas.

The arm motor task elicited strong activations of the affected hand-knob area (LI M1 = 0.42) and healthy SMA (LI SMA = −1), as shown in Figure 2 and Table 3. The leg motor task elicited strong activations of both paracentral lobules (LI M1 = −0.22) and healthy SMA (LI SMA = −1). After surgery, activations shifted to the intact hemisphere, engaging the peri-rolandic cortex and a mesial area including the SMA for both paretic arm and leg. fMRI activations remained in similar locations at the different follow-ups.

**Table 3:** Percentage of fMRI activations of the motor network, comprising the affected primary motor area (aM1) and the affected SMA (aSMA), and the unaffected homologous areas (uM1, uSMA), with corresponding Laterality Index (LI) for patients and control subjects.

**Participants**	**aM1**	**aSMA**	**uM1**	**uSMA**	**uM1+uSMA**	**M1**	**SMA**	**M1+SMA**
	**% fMRI motor activation—ARM**					**LI**		
Case 1	0.47	0.00	0.19	0.35	0.53	0.42	−1.00	−0.07
Case 2	0.30	0.26	0.11	0.33	0.44	0.47	−0.11	0.12
Case 3	0.29	0.00	0.60	0.11	0.71	−0.34	−1.00	−0.41
Case 4	0.27	0.41	0.07	0.25	0.32	0.59	0.24	0.36
Controls	0.74	0.04	0.15	0.07	0.22	0.67	−0.22	0.57
	**% fMRI motor activation—LEG**					**LI**		
Case 1	0.34	0.00	0.53	0.13	0.66	−0.22	−1.00	−0.32
Case 2	0.54	0.20	0.08	0.19	0.26	0.75	0.03	0.47
Case 3	0.37	0.00	0.28	0.35	0.63	0.13	−1.00	−0.26
Case 4	0.38	0.06	0.47	0.09	0.56	−0.10	−0.17	−0.11

*Negative LI indicates unaffected side*.

#### DTI

Before surgery, the CST was reconstructed in both hemispheres, although the ipsi-lesional tract was more reduced; there were no inter-hemispheric fibers, as the corpus callosum was severely atrophic (Figure 2). After surgery the right CST was no longer visible and the right somatosensory tract was recognized only between the brainstem and the thalamus. A quantitative analysis of FAz values at the pons showed that the CST was more lateralized than in control subjects (LI FAz = −0.23 vs. LI FAz = −0.03, respectively). After surgery lateralization of CST increased over time, reaching extreme values 3 years after disconnection (Table 4).

**Table 4:** Lateralization Index (LI) of FA values at pons level in patients before and after surgery at different follow-ups, and in control subjects.

	**Time**	**FA**	**FAz**
Case 1 PRE	12 days	−0.14	−0.23
Case 1 POST1	6 months	−0.21	−0.60
Case 1 POST2	1 year	−0.22	−0.60
Case 1 POST3	3 years	−0.48	−0.69
Case 2 PRE	3 days^*^	−0.20	−0.35
Case 2 POST1	18 months	−0.37	−0.55
Case 2 POST2	3 years	−0.35	−0.64
Case 3 PRE	10 days	−0.25	−0.76
Case 3 POST 1	2 years	−0.24	−0.77
Case 3 POST2	3 years	−0.17	−0.60
Case 4 PRE	3 days	−0.77	−0.77
		**Mean (sd)**	**Mean (sd)**
Control subjects		−0.02 (0.05)	−0.03 (0.04)

FAz refers to FA values in the z direction. Negative values indicate lateralization toward the unaffected hemisphere for patients and toward the left hemisphere for controls. Patients differed significantly from the control group at all sessions (>2 SD).

* *The patient was imaged on a 1.5 Tesla scanner for this session only*.

### Case 2

The patient (age range 30–35 years) suffered from a severe brain trauma at the age of 6 months, developing medically-refractory epilepsy with left side tonic and clonic seizures some months later. She had left hemiparesis, more prominent in the upper limb, with limited hand grasping function and no fine finger movement (Table 2). MRI showed a right frontal malacic area involving frontal convolutions, partially sparing the motor cortex, and also a left, smaller, frontal malacic area. The right cerebral peduncle was markedly atrophic (Figure 1). Despite the extension of the right malacic area, CST was recognizable also in the posterior limb of the internal capsule. She underwent a right fronto-central cortectomy and a disconnection of fronto-central region and CST. After surgery the MRI showed removal of the upper part of the precentral cortex, including the lobulus paracentralis; the right CST was no longer recognizable. Approximately 3 years after surgery, the patient was seizure free (Engel class Ia). Motor function initially worsened, but after 18 months recovered, and at 3 years after surgery was unchanged for both limbs from baseline (Table 2).

#### fMRI

The arm motor task elicited activations of the precentral gyrus bilaterally, with stronger activation of the ipsi-lesional hand-knob area (LI M1 = 0.47). The SMA was activated bilaterally (LI SMA = −0.11), as reported in Figure 2 and Table 3. The foot motor task elicited strong activation of the ipsi-lesional paracentral lobule and, marginally, of the area anterior to the contro-lesional paracentral lobule (LI M1 = 0.75). The healthy SMA was slightly activated (LI SMA = 0.03). After surgery, activations shifted to the healthy hemisphere. For the arm, fMRI activations engaged a portion of the pre- and post-central gyri, and SMA with the premotor area. Interestingly, activation of the paretic leg appeared anteriorly and posteriorly to the paracentral lobule; this was activated by the healthy leg, resulting in activations that were topographically neighboring (healthy side movement not shown). Generally, significant fMRI activations remained in approximally the same location at the different follow-ups.

#### DTI

Before surgery, the CST was reconstructed in both hemispheres, although the affected tract was reduced, and inter-hemispheric fibers connecting homologous motor regions were visualized (Figure 2). After surgery, the right CST was no longer visible and the right somatosensory tract was recognized only between the brainstem and the thalamus. A quantitative analysis of FAz values at the pons showed that the affected CST was reduced compared to control subjects (LI FAz = −0.35 vs. LI FAz = −0.03, respectively). After surgery, lateralization of CST increased strongly over time (Table 4).

### Case 3

The patient (age range 35–40 years) suffered from perinatal asphyxia, developing drug-refractory epilepsy at 6 years with right side tonic seizures. She had right hemiparesis, more prominent in the upper limb, with control of hand grasping but no fine finger movement (Table 2). MRI showed a large left frontal malacic area partially extending to the rolandic cortex, sparing only the upper part of the motor strip (Figure 1). The posterior limb of the internal capsule and the CST were not recognizable and the left cerebral peduncle was markedly atrophic. The patient underwent a left frontal lobectomy and disconnection of about two thirds of the body of the corpus callosum. After surgery the MRI demonstrated removal of the frontal convolutions, including the motor cortex, and disconnection of approximately the anterior half of the corpus callosum. Four years after surgery, the patient was free of seizures (Engel class Ia). Motor functions were overall unchanged 3 years after surgery (Table 2).

#### fMRI

**Figure 3:**
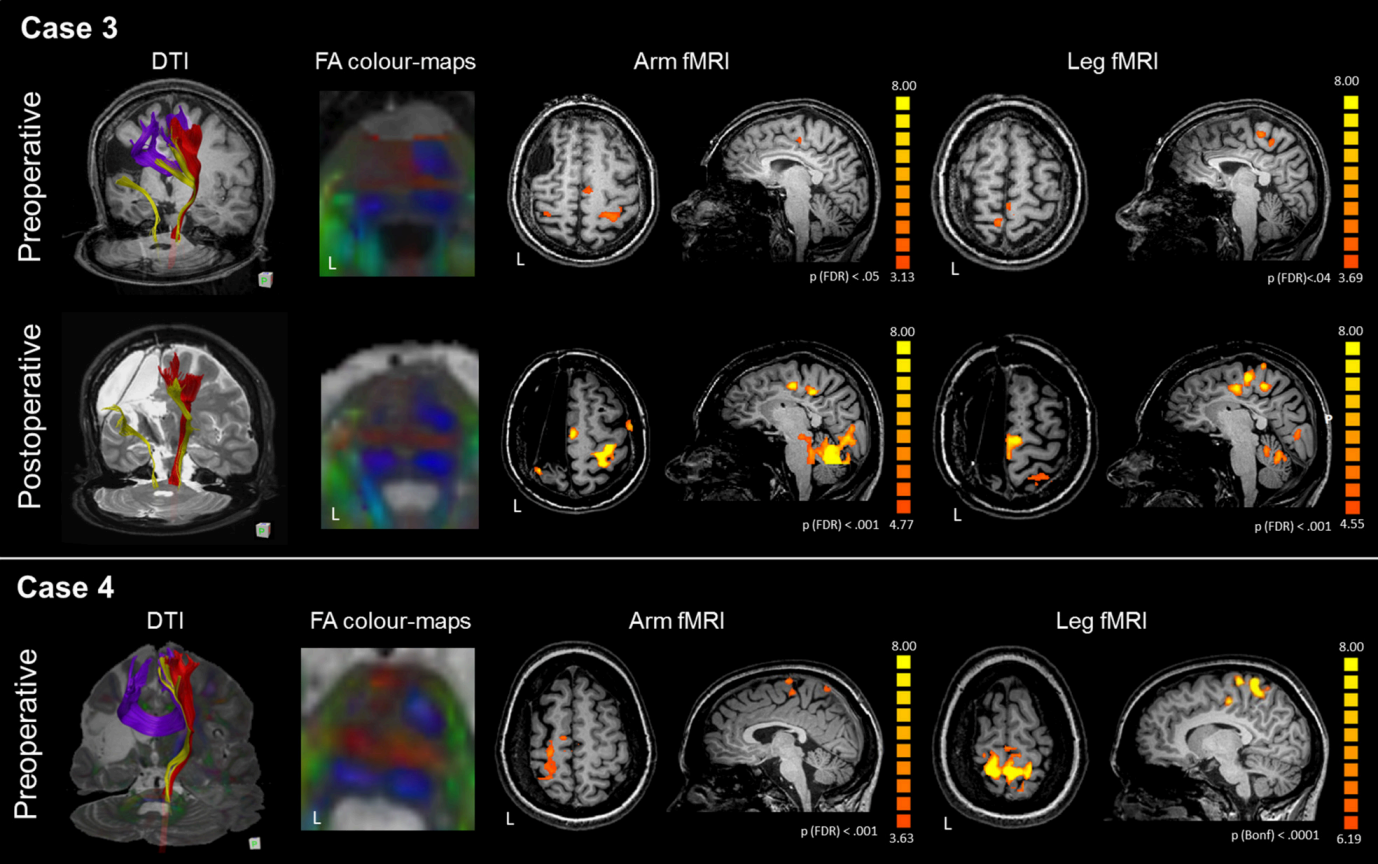
DTI-tractography and fMRI tasks of arm and leg movement of the preoperative and postoperative assessment of Case 3 and Case 4. Case 3 presented with a large right frontal malacic area. In the affected hemisphere, DTI-tractography did not reconstruct the CST (red), but revealed a thin somatosensory tract (yellow) and inter-hemispheric fibers connecting homologous motor regions. After surgery the interhemispheric fibers were no longer visible. FA color-maps at the pons showed absence of CST in the left hemisphere even before surgery. Before surgery, fMRI activations of the paretic arm and leg were generally bilateral, with involvement of intact SMA. After surgery, activations shifted to the intact hemisphere with a strong engagement of the SMA. Case 4 presented with a large left malacic area. In the affected hemisphere, DTI-tractography did not reconstruct the CST (red), nor the somatosensory tract (yellow), while inter-hemispheric fibers connecting homologous motor regions were observed. FA color-maps at the pons showed absence of CST in the left hemisphere even before surgery. fMRI activations of the paretic arm and leg strongly involved the affected motor cortex, and the intact SMA. For technical problems the patient could not be scanned with MRI after surgery.

The arm motor task elicited activations of the precentral gyrus bilaterally, with stronger activation of the intact hand-knob area (LI M1 = −0.34), as reported in Figure 3 and Table 3. The SMA was activated only in the healthy hemisphere (LI SMA = −1). The leg motor task elicited activations of the paracentral lobule bilaterally (LI M1 = 0.13) and healthy SMA (LI SMA = −1). After surgery, fMRI motor activations of the paretic hand engaged a significant portion of the healthy hand-knob area, while in the affected hemisphere a small cluster of activation was still visible in the somatosensory area. For the paretic leg, fMRI activations engaged an area anterior to the paracentral lobule and SMA in the healthy hemisphere. These fMRI activations remained stable at the different follow-ups.

#### DTI

In the affected hemisphere DTI-tractography did not reconstruct the left CST, while a reduced tract from the left posterior pons toward the parietal lobe was visible. Inter-hemispheric fibers connecting homologous motor regions were observed (Figure 3). After surgery the interhemispheric fibers were no longer visible. A quantitative analysis of FAz values at the pons showed a strong lateralization of CST before surgery (LI FAz = −0.76) and after it (Table 4).

### Case 4

The patient (age range 25–30 years) suffered from an ischemic stroke at 2 years, developing drug-refractory epilepsy at 12 years with right-sided tonic seizures. She had right hemiparesis with control of shoulder and elbow, but not hand and mild paresis of lower limb (Table 2). MRI showed a large left malacic area involving the inferior and lateral portion of the motor cortex, left CST, internal capsule and left brainstem (Figure 1). The patient underwent a left fronto-parietal disconnective surgery with resection of fronto-parietal areas. After surgery the MRI showed the presence of a drill fragment under the skull giving strong artifacts and preventing to make further MRI exams. Postoperatively, the patient was not seizure free (Engel IV). Motor functions were unchanged at 6 and 12 months after surgery.

#### fMRI

The arm motor task elicited strong activations of the hand-knob area of the affected hemisphere (LI M1 = 0.59), as reported in Figure 3 and Table 3. The SMA was activated bilaterally (LI SMA = 0.24). The leg motor task elicited bilateral activations of the paracentral lobule (LI M1 = −0.10). The SMA was activated only marginally (LI SMA = −0.17).

#### DTI

In the affected hemisphere DTI-tractography did not reconstruct the left CST, nor the somatosensory tract. Inter-hemispheric fibers connecting homologous motor regions were observed (Figure 3). A quantitative analysis of FAz values at the pons showed a strong lateralization of CST before surgery (LI FAz = −0.77) (Table 4).

## Discussion

We studied with fMRI and DTI the motor functions of four patients undergoing tailored hemispheric surgery for drug-refractory epilepsy. Patients were rather homogeneous for some clinical features: they all suffered from a damage acquired early in life (<2 years), were operated in adulthood (age range: 25–39 years), and surgery removed the cortical motor areas and disconnected the CST, substantially depriving the affected hemisphere of motor functions. Nevertheless, after surgery, patients maintained their elementary motor abilities, being able to move their paretic arm and walk independently. By tracking recovery of motor functions before and after surgery with fMRI and DTI, we observed three main results: First, before surgery patients were characterized for the presence/absence of the affected CST, as revealed by DTI-tractography and FA-maps. Second, lateralization observed with fMRI and DTI was useful to understand how motor circuits were organized and assess the risks of postoperative deficits, especially when results were confirmed by both techniques. Third, the reorganization of motor network after damage and subsequent surgery can involve both primary motor areas as well as associative areas and continue for a long time (>1 year) after disconnection.

### DTI results

DTI-tractography revealed the presence of the affected CST in two cases. In particular, for Case 1 and Case 2 the CST was reconstructed in both sides with the affected CST reduced compared to the contralateral one. The surgical disconnection of CST resulted in a drop of FAz values, indicating a degeneration of fibers in the affected hemisphere for both patients. Postoperatively, Case 1 with an atrophic corpus callosum showed worsening of paretic limbs, while Case 2 maintained motor functions. By contrast, for Case 3 and Case 4, DTI-tractography did not reconstruct the CST in the affected hemisphere. The FAz values were strongly asymmetric before and after disconnection, confirming that the CST was substantially functional in one side only. After surgery, motor functions of upper and lower limbs remained clinically stable in both patients.

### Lateralization of motor pathways and fMRI activations

The lateralization of motor networks and the consistency of DTI and fMRI findings is critical in the preoperative assessment of these four patients, as reorganization of motor areas in the healthy hemisphere could preserve motor functions after surgery.

When the CST is present only in the healthy hemisphere and fMRI activations are lateralized to the healthy side, it is likely that motor functions will be maintained or even will improve after surgery. This is the case of Patient 3, with the CST reconstructed only in the intact hemisphere, FA-maps strongly asymmetric and the fMRI motor network substantially lateralized toward the healthy hemisphere. Her motor functions did not change after disconnection. This ipsilateral reorganization, observed with both techniques, represents the best condition for recovery after surgery. It is also possible that the CST is present only in the healthy hemisphere but fMRI activations are bilateral or even lateralized to the affected hemisphere. This is the case of Patient 4, with only one CST, markedly asymmetric FAz values and activations bilaterally for the leg and lateralized to the damaged hemisphere for the arm. Her motor functions were very limited, especially for the upper limb, and did not change after surgery. A possible explanation of this case is that the intact hemisphere contributed decisively to the movement of the paretic limb, receiving input from the affected motor areas via corpus callosum and this would account for the presence of bilateral fMRI activations. Similar findings were observed also by Pilato et al. (21) and Staudt et al. (6). Moreover, as suggested by Wang et al. the ipsilateral pathway might have been partially inhibited by the activity of the affected hemisphere in the preoperative state, and the surgical disconnection might have unmasked the contribution of the ipsilateral tract (20). In this case, fMRI activations were less consistent with DTI lateralization.

By contrast, it is possible that motor pathways are bilateral: if CST is reconstructed in the affected hemisphere and fMRI activations are bilateral, in theory the risk of motor decline should be higher. In practice, Case 2 did not worsen: quantifying the lateralization of the motor network, DTI and fMRI revealed a moderate lateralization. It is possible that the motor pathways of the intact hemisphere contributed strongly to the paretic arm already before surgery, and that disconnective surgery might have facilitated ipsilateral motor control of limb function through disinhibition of the ipsilateral tract, as proposed by Wang et al. (20). The patient suffered from a severe (multilobar) lesion at 6 months: this early lesion might have triggered compensatory mechanisms in the healthy hemisphere and this would explain the effective reorganization observed even if surgery occurred in adulthood. This case suggests that the presence of the CST and a quantitative or semi-quantitative (19) analysis of its integrity with FA-maps can better assess the risks of postoperative deficits.

The other patient with a more bilateral pattern was Case 1 and she worsen postoperatively. In this case both techniques showed a more bilateral pattern compared to the other cases. The corpus callosum was strongly damaged by the hemispheric lesion at 1 year of age, and probably not functional to connect homologous motor regions. It is possible that the intact hemisphere, not receiving motor inputs from the contralateral hemisphere, could not develop a motor representation, a “copy” of the paretic limb and, after surgery, could not contribute significantly to the movement of the affected limb (31, 32). This case suggests that a more bilateral pattern, measured with fMRI and DTI, places the patient at risks of postoperative deficits. Generally, in these patients the ipsilateral involvement represents the only possibility of recruitment after surgery. Therefore, the more the ipsilateral motor pathway is involved, the greater the likelihood of maintaining motor functions after surgery.

### Reorganization of motor areas

Another critical issue refers to the reorganization of motor areas, whether it involves primary or associative areas and whether modifications continue over time. From studies after hemispherectomy, it is known that movement of paretic limbs is associated to the intact hemisphere (15, 16), but it is not known whether this pattern was the same even before surgery. In this study our four patients displayed stronger activations of the intact motor areas, in particular the SMA, compared to control subjects, even before surgery (see Table 3). After surgery, fMRI of the paretic limbs show that the SMA was always engaged, while the primary motor area was only weakly activated in some sessions, like in Case 1 for arm movement, and in Case 2 and Case 3 for leg movement. Postoperative fMRI activations remained generally in similar locations at the different follow-ups. In addition, SMA activation had generally the same location in preoperative and postoperative sessions: it is likely that SMA contributed significantly to the movement of the paretic limb after the first, early damage and then after surgery. These results, derived only from three patients (Case 4 could not be imaged after surgery) are in line with fMRI studies showing that the intact SMA contributes to the reorganization of motor network (15, 33, 34), and support the hypothesis that also the SMA can exert motor control (15, 16). In our cases reorganization of motor circuits was not restricted to a few months, but changes in the integrity of CST were progressive at least 1 year after surgery, as shown by FA values before and after surgery at different follow-ups (Table 4).

There are other considerations. Regarding the paretic leg movement, our patients maintained lower limb function and locomotor control after surgery, except for Case 1 who slightly worsened. This is in line with previous studies showing that hand movements, especially finger movements, are subject to a stronger modulation of cortical control, while locomotion is more under the control of subcortical regions such as brainstem, cerebellum and spinal neuronal circuits (12, 35–37). In this study it is difficult to establish whether the re-organized motor circuit involves the ipsilateral corticospinal pathway or extra-pyramidal pathways. It is likely that the ipsilateral pathway is involved, as suggested by previous studies (6, 7), but it is not possible to establish its contribution with respect to subcortical or spinal mechanisms. These results might be useful in the framework of rehabilitation: identifying the motor pathways and cortical areas exerting motor control of the paretic limbs can be relevant to reinforce these circuits with adequate rehabilitation strategies (11).

This work has a number of limitations. First, the sample size is small and Case 4 was not imaged after surgery, therefore inferences should be taken cautiously. Second, the presence of CST was assessed anatomically with DTI and with radiological observations, but not functionally with TMS. However, TMS was not applied in these patients for the risk of inducing epileptic seizures, even if the risk is low (38). Third, different DTI sequences were used during the years and this could affect longitudinal quantitative evaluations. However, the analyses were performed calculating relative measures, i.e., lateralization indexes. Fourth, control subjects did not perform the leg motor task. However, we focused on the hand/arm motor task, which is generally more investigated in the literature (37).

## Conclusions

fMRI and DTI were useful non-invasive tools to investigate the mechanisms of motor plasticity in four patients candidate to disconnection surgery (20, 39, 40). The results of our small sample show that the brain motor network has a redundancy of pathways (ipsilatereal and contralateral CST, primary motor cortex and associative motor areas such as SMA) which can be used as compensatory mechanisms. In particular, our results suggest that the more the patients engage the intact hemisphere, the greater are the chances of maintaining elementary motor functions after disconnection surgery. In particular, in these four patients, DTI-tractography with quantification of FA-maps was useful to assess the lateralization of the motor network. Finally, structural alterations of motor circuits are progressive and long lasting after surgery.

## Author contributions

CR and FG conceived and designed the study. All authors acquired and analyzed the data. CR and FG drafted the manuscript and all authors approved the final version. FV and FG contributed equally.

### Conflict of interest statement

The authors declare that the research was conducted in the absence of any commercial or financial relationships that could be construed as a potential conflict of interest. The reviewer MG and handling Editor declared their shared affiliation.
