# Supplementary material for: Tracking the Re-organization of Motor Functions After Disconnective Surgery: A Longitudinal fMRI and DTI Study
Source: Front Neurol. 2018 Jun 5;9:400. doi: 10.3389/fneur.2018.00400 (PMC5996100; doi:10.3389/fneur.2018.00400)
Supplement: Supplementary file 1 [file Table_1.DOC]

|  |  | **PRE** | **POST 1** | **POST 2** | **POST 3** |
| --- | --- | --- | --- | --- | --- |
| **Case 1** | Time | 12 days | 6 months | 1 year | 3 years |
|  | TR/TE | 8100/75 | *u.* | *u.* | *u.* |
|  | flip angle | 90 | *u.* | *u.* | *u.* |
|  | b values | 0, 1000 | *u.* | *u.* | *u.* |
|  | N directions | 64 | *u.* | *u.* | *u.* |
|  | N slices | 70 | *u.* | *u.* | *u.* |
|  | FOV | 224 224 153.8 | *u.* | *u.* | *u.* |
|  | voxel | 2 2 2 | *u.* | *u.* | *u.* |
| **Case 2** | Time | 3 days* | 18 months | 3 years |  |
|  | TR/TE | 9300/100 | 8100/75 | *u.* |  |
|  | flip angle | 90 | 90 | *u.* |  |
|  | b values | 0, 1200 | 0,1000 | *u.* |  |
|  | N directions | 64 | 64 | *u.* |  |
|  | N slices | 64 | 70 | *u.* |  |
|  | FOV | 256 256 147.2 | 224 224 153.8 | *u.* |  |
|  | voxel | 2.29 2.29 2.3 | 2 2 2 | *u.* |  |
| **Case 3** | Time | 10 days | 2 years | 3 years |  |
|  | TR/TE | 6078/75 | 8100/75 | *u.* |  |
|  | flip angle | 90 | 90 | *u.* |  |
|  | b values | 0, 1000 | 0, 1000 | *u.* |  |
|  | N directions | 64 | 64 | *u.* |  |
|  | N slices | 50 | 70 | *u.* |  |
|  | FOV | 224 224 134.8 | 224 224 153.8 | *u.* |  |
|  | voxel | 2.33 2.33 2.50 | 2 2 2 | *u.* |  |
| **Case 4** | Time | 3 days |  |  |  |
|  | TR/TE | 8092/75 |  |  |  |
|  | flip angle | 90 |  |  |  |
|  | b values | 0, 1000 |  |  |  |
|  | N directions | 64 |  |  |  |
|  | N slices | 70 |  |  |  |
|  | FOV | 224 224 153.8 |  |  |  |
|  | voxel | 2 2 2 |  |  |  |

Supplementary Table 1. Information about the DTI sequence used in the different sessions for each patient. PRE refers to the preoperative assessment; POST 1/2/3 refer to the postoperative follow-ups. Time refers to the interval between imaging and surgery. '*u.*' refers to unchanged sequence parameters with respect to the previous time point. * The patient was imaged on a 1.5 Tesla scanner for this session only.
